# Data from a cross-sectional study on Apolipoprotein E (*APOE*-ε4) and snoring/sleep apnea in non-demented older adults

**DOI:** 10.1016/j.dib.2015.09.014

**Published:** 2015-09-30

**Authors:** Angeliki Tsapanou, Nikolaos Scarmeas, Yian Gu, Jennifer Manly, Nicole Schupf, Yaakov Stern, Sandra Barral

**Affiliations:** a Cognitive Neuroscience Division, Columbia University Medical Center, New York, USA; b The Gertrude H. Sergievsky Center, Columbia University Medical Center, New York, USA; c The Department of Neurology, College of Physicians and Surgeons, Columbia University Medical Center, New York, USA; d National and Kapodistrian University of Athens Medical School, Greece

**Keywords:** Apolipoprotein E, Snoring, Sleep apnea, Elderly

## Abstract

In the present data, we provide the details of the cross-sectional study, from the Washington Heights-Inwood Community Aging Project (WHICAP) that examined the association between Apolipoprotein E (*APOE*-ε4) and snoring/sleep apnea. A total of 1944 non-demented older adults constituted our sample. Sleep dysfunction was measured using sleep categories derived from the Medical Outcomes Study Sleep Scale. Stratified analyses were conducted in order to examine the association between *APOE*-ε4 and sleep variables by ethnic group. For further analyses and enhanced discussion, see “*Examining the association between Apolipoprotein E (APOE) and self-reported sleep disturbances in non-demented older adults*” by Tsapanou et al. (2015) [1].

**Specifications Table**

**Table:** 

Subject area	Neuropsychology, Neurology, Genetics
More specific subject area	Neurogenetics
Type of data	Questionnaire, Tables
How data was acquired	Quantitative data of 1944 non-demented elderly, using a self-reported sleep questionnaire and their *APOE*-ε4 status
Data format	Raw data, Analyzed
Experimental factors	*APOE*-ε4 and sleep questions were the main variables used. Age, sex, ethnicity, years of education, and body mass index were used as covariates.
Experimental features	Characterization of the *APOE*-ε4 status
Data source location	New York, New York, USA
Data accessibility	Data is in this article

**Value of the data**

• Identifying the sleep measures associated with *APOE*-ε4.
• Providing novel information about the differences among different ethnic groups.
• Providing the frequencies of the *APOE* alleles in a large sample of older adults.

## 1 Data, experimental design, materials and methods

In the present data, we present the details of the cross-sectional study that examined the association between Apolipoprotein E (*APOE*-ε4) and snoring/sleep apnea in a large group of older adults. We provide the demographic characteristics of our sample, the frequencies of the *APOE* alleles in our population, the sleep scale we used, and the results of the association analyses between *APOE*-ε4 and sleep variables stratified by ethnic group [1].

### 1.1 Sample collection

Participants were drawn from the Washington Heights-Inwood Community Aging Project (WHICAP) at Columbia University Medical Center [2,3]. WHICAP is a community based research study designed to identify risk factors and biomarkers for aging and Alzheimer׳s disease in a multi-ethnic cohort that includes Whites, African-Americans, Caribbean-Hispanics, and Other [4]. All participants were over 65 years old, and non-demented at the time of the evaluation. For details, see supplementary Table 1.

### 1.2 Sleep measures

Sleep quality was assessed using the Sleep Scale from the RAND Medical Outcomes Study. This scale is a self-report 12-item questionnaire [5,6]. Each of the questions has a possible rating of 0-6, based on the frequency of the sleep problem. Using the sleep questionnaire manual [6], we used the five clustered sleep categories to define our analyses phenotype: 1. Sleep disturbance, 2. Snoring, 3. Sleep short of breath/ awaking with a headache, 4. Sleep adequacy, and 5. Daytime somnolence. Additionally, categories 2 and 3 were combined into a single variable ‘sleep apnea’ (see Supplementary Fig. 1).

### 1.3 *APOE* genotyping

WHICAP participants were *APOE* genotyped as previously described [7]. *APOE* genotypes were transformed into a dichotomous trait based on the number of *APOE*-ε4 alleles: 0 if the individual does not carry any copy of the ε4 allele (non-ε4 carriers) or 1 if the individual carries 1 or 2 copies of the ε4 allele (ε4 carriers). Carriers of ε2ε4 alleles were not included in the initial sample due to the opposite effect of these two alleles [8–10]. More details about the *APOE* genotyping can be found in previous studies [7]. For the frequencies of the *APOE* alleles, see supplementary Table 2.

### 1.4 Analysis

All statistical analyses were performed using SPSS 22 (SPSS, Chicago, Illinois). Nominally significant *p* values were defined as *p*<0.05. Unadjusted Linear Regression Analyses: We used linear regression models with *APOE*-ε4 at baseline evaluation as the predictor and the previously described sleep score as the independent variable. Adjusted Linear Regression Analyses: Secondary analyses were performed adjusting for: age, sex, ethnicity, education, and body mass index (BMI). Ethnicity was ascertained based on self-report using the format of the 1990 census [11]. Participants were then assigned to one of the three groups: White, African-American, and Caribbean-Hispanic. To further examine any possible differences among the ethnic groups, we stratified the sample and perform analyses within each ethnic group independently (Whites *n*= 431, African Americans *n*=465, and Caribbean-Hispanics n= 1048) (see Supplementary Table 3).

## Footnotes

Appendix A Supplementary data associated with this article can be found in the online version at doi:10.1016/j.dib.2015.09.014.
