# Supplementary material for: Data from a cross-sectional study on Apolipoprotein E (APOE-ε4) and snoring/sleep apnea in non-demented older adults
Source: Data Brief. 2015 Sep 30;5:351–3. doi: 10.1016/j.dib.2015.09.014 (PMC4602351; doi:10.1016/j.dib.2015.09.014)
Supplement: Supplementary file 1 — Supplementary material [file mmc1.docx]

| **Elements of financial/ personal conflicts** | **Angeliki Tsapanou** | **Yian Gu** | **Sandra Barral** | **Nicole Schupf** | **Jennifer Manly** | **Nikolaos Scarmeas** | **Yaakov Stern** |
| --- | --- | --- | --- | --- | --- | --- | --- |
| Affiliation | Columbia University | Columbia University | Columbia University | Columbia University | Columbia University | Columbia University | Columbia University |
| Grands/funds | no | no | no | no | no | no | no |
| Honoraria | no | no | no | no | no | no | no |
| Speaker forum | no | no | no | no | no | no | no |
| Consultant | no | no | no | no | no | no | no |
| Stocks | no | no | no | no | no | no | no |
| Royalties | no | no | no | no | no | no | no |
| Expert testimony | no | no | no | no | no | no | no |
| Board member | no | no | no | no | no | no | no |
| Patents | no | no | no | no | no | no | no |
| Personal relationship | no | no | no | no | no | no | no |

**Conflict of interest disclosures:**
