# Supplementary material for: Data from a cross-sectional study on Apolipoprotein E (APOE-ε4) and snoring/sleep apnea in non-demented older adults
Source: Data Brief. 2015 Sep 30;5:351–3. doi: 10.1016/j.dib.2015.09.014 (PMC4602351; doi:10.1016/j.dib.2015.09.014)
Supplement: Supplementary file 2 — Supplementary material [file mmc2.zip › Supplementary table 2.docx]

Supplementary table 2

# Frequencies of the *APOE* alleles.

| APOE | N | % |
| --- | --- | --- |
| 2-2 | 17 | 0.8 |
| 2-3 | 292 | 13.5 |
| 3-3 | 1,349 | 62.5 |
| 3-4 | 481 | 22.3 |
| 4-4 | 20 | 0.9 |
|  | 2,159 | 100.0 |
