# Supplementary material for: Data from a cross-sectional study on Apolipoprotein E (APOE-ε4) and snoring/sleep apnea in non-demented older adults
Source: Data Brief. 2015 Sep 30;5:351–3. doi: 10.1016/j.dib.2015.09.014 (PMC4602351; doi:10.1016/j.dib.2015.09.014)
Supplement: Supplementary file 2 — Supplementary material [file mmc2.zip › Supplementary table 3.docx]

Supplementary table 3

| **Sleep variable** |  | |  | | |  |  | | **Correlation with *APOE*-ε4** | | |  |
| --- | --- | --- | --- | --- | --- | --- | --- | --- | --- | --- | --- | --- |
|  | **Whites** | | | | | | **African- Americans** | | | | | **Caribbean-Hispanics** |
|  | **B** | **SE** | | **Beta** | ***p*** | | **B** | **SE** | | **Beta** | ***p*** | **B SE Beta *p*** |
|  |  |  | |  |  | |  |  | |  |  |  |
| **Snoring** | 0.01 | 0.02 | | 0.03 | 0.592 | | -0.03 | 0.02 | | -0.09 | 0.082 | -0.02 0.01 -0.10 **0.004** |
| **Sleep apnea** | 0.00 | 0.01 | | 0.01 | 0.923 | | -0.02 | 0.02 | | -0.08 | 0.132 | -0.02 0.01 -0.08 **0.021** |

Association analyses between *APOE*-ε4 and sleep variables stratified by ethnic group.
