# Supplementary material for: Data from a cross-sectional study on Apolipoprotein E (APOE-ε4) and snoring/sleep apnea in non-demented older adults
Source: Data Brief. 2015 Sep 30;5:351–3. doi: 10.1016/j.dib.2015.09.014 (PMC4602351; doi:10.1016/j.dib.2015.09.014)
Supplement: Supplementary file 2 — Supplementary material [file mmc2.zip › Supplementary table 1.docx]

Supplementary table 1

# Demographic characteristics of the *APOΕ*-ε4 carriers and non-ε4 carries.

| **Characteristics** | ***APOΕ*-ε4 carriers** | ***APOΕ*-ε4 non-carriers** | **Total** |
| --- | --- | --- | --- |
| Age at visit (years), Mean(SD) | 79 (6.6) | 80 (6.7) | 80 (6.7) |
| Education (years), Mean(SD) | 10 (5.1) | 10 (5.0) | 10 (5.0) |
| Gender, N (%) |  |  |  |
| Female | 340 (70.2) | 997 (68.3) | 1337 (68.8) |
| Male | 144 (29.8) | 463 (31.7) | 607 (31.2) |
| Ethnicity, N (%) |  |  |  |
| White | 86 (17.8) | 345 (23.6) | 431 (22.2) |
| African-American | 168 (34.7) | 297 (20.3) | 465 (23.9) |
| Caribbean-Hispanic | 230 (47.5) | 818 (56.0) | 1048 (53.9) |
| BMI, Mean(SD) | 28.63 (5.6) | 29.03 (5.5) | 28.93 (5.5) |
| Total, N (%) | 484 (24.9) | 1460 (75.1) | 1. (100) |
