# Supplementary material for: Data from a cross-sectional study on Apolipoprotein E (APOE-ε4) and snoring/sleep apnea in non-demented older adults
Source: Data Brief. 2015 Sep 30;5:351–3. doi: 10.1016/j.dib.2015.09.014 (PMC4602351; doi:10.1016/j.dib.2015.09.014)
Supplement: Supplementary file 2 — Supplementary material [file mmc2.zip › Supplementary figure 1.docx]

Supplementary figure 1

Sleep Scale from the Medical Outcomes Study [6]

1. How long did it usually take for you to fall asleep during the past 4 weeks?

(Circle One)

0-15 minutes…………..…..1

16-30 minutes……………..2

31-45 minutes……………..3

46-60 minutes……………..4

More than 60 minutes …….5

_________________________________________________________

2. On the average, how many hours did you sleep each night during the past 4 weeks?

Write in number of hours per night:

**_________________________________________________________**

How often during the past 4 weeks did you:

3. Feel that your sleep was not quiet (moving restlessly, feeling tense, speaking, etc., while sleeping)?

4. Get enough sleep to feel rested upon waking in the morning?

5. Awaken short of breath or with a headache?

6. Feel drowsy or sleepy during the day?

7. Have trouble falling asleep?

8. Awaken during your sleep time and have trouble falling asleep again?

9. Have trouble staying awake during the day?

10. Snore during your sleep?

11. Take naps (5 minutes or longer) during the day?

12. Get the amount of sleep you needed?

*Possible answers: 1= All of the time, 2= Most of the time, 3= A good bit of the time, 4= some of the time, 5= A little of the time, 6= none of the time, -1= Not asked, -2= Too impaired to respond, -3= Refused*

Sleep categories

1. Sleep disturbance (included questions: ‘How long did it usually take for you to fall asleep during the past 4 weeks?’, ‘Feel that your sleep was not quiet (moving restlessly, feeling tense, speaking, etc.) while sleeping?’, ‘Have trouble falling asleep?’, ‘Awaken during your sleep time and have trouble falling asleep again?’),

2. Snoring (‘Snore during your sleep?’),

3. Sleep short of breath/ headache (‘Awaken short of breath or with a headache?’),

4. Sleep adequacy (‘Get enough sleep to feel rested upon waking in the morning?’, ‘Get the amount of sleep you needed?’)

5. Daytime somnolence (‘Feel drowsy or sleepy during the day?’, ‘Have trouble staying awake during the day?’, ‘Take naps (5 minutes or longer) during the day?).

Additionally, categories 2 and 3 were combined into a single variable ‘sleep apnea’

Copyright, 1986, RAND
